# Exercise‐Induced Desaturation in COPD Patients Postexacerbation: Impact of Different Definitions on Prevalence Rates in a Rehabilitation Setting

**DOI:** 10.1111/crj.70199

**Published:** 2026-05-31

**Authors:** Beatrice Salvi, Mara Paneroni, Carla Simonelli, Rodolfo Murgia, Francesca Dal Negro, Michele Vitacca

**Affiliations:** ^1^ Respiratory Rehabilitation Unit Istituti Clinici Scientifici Maugeri IRCCS Lumezzane Brescia Italy; ^2^ Respiratory Rehabilitation Unit Istituti Clinici Scientifici Maugeri IRCCS Montescano Pavia Italy; ^3^ Respiratory Rehabilitation Unit Istituti Clinici Scientifici Maugeri IRCCS Veruno Novara Italy

**Keywords:** 6MWT, COPD, EID prevalence, exercise‐induced desaturation, oxygen therapy, SpO_2_

## Abstract

**Background:**

Exercise‐induced oxygen desaturation (EID) refers to a significant decrease in oxygen saturation (SpO_2_) during physical exertion, commonly observed in chronic obstructive pulmonary disease (COPD) patients. Definitions of EID vary widely between studies, leading to difficulties in comparing results and inconsistencies in clinical practice.

**Objective:**

The objectives of this study were to evaluate (a) the different definitions of EID reported in the literature, (b) the prevalence of EID during the 6‐min walking test (6MWT) in a cohort of COPD patients undergoing pulmonary rehabilitation (PR), and (c) the agreement between the definitions.

**Methods:**

A scoping review was conducted to assess existing EID definitions. The prevalence of EID in a cohort of postexacerbation COPD patients performing the 6MWT at admission to PR was retrospectively evaluated based on selected definitions. Agreement between definitions was assessed using Fleiss' kappa.

**Results:**

Of the 244 articles reviewed, 62 met the inclusion criteria, and 23 definitions of EID were identified. The seven most cited definitions found were applied to 954 patients who completed the 6MWT at admission. The most common criterion was a ≥ 4% decrease in SpO_2_ during walking. The prevalence of EID varied between 15.6% and 38.7% depending on the criterion used. The overall agreement between the definitions was good, with a Fleiss kappa of 0.718.

**Conclusions:**

In a large COPD cohort recovering from exacerbation, EID prevalence varied significantly depending on the definition used. Although good agreement was found between the criteria, the most conservative definition identified EID in only 15% of patients. Future studies will be necessary to determine which definition is most appropriate, predictive for outcomes, and useful for prescribing oxygen under effort.

## Graphical abstract

As there is no standardized definition of exertional desaturation, the frequency with which it is reported varies widely. Although researchers currently agree to a good extent (*κ* = 0.718 according to Fleiss' kappa), a single, consistent definition is necessary in order to reliably capture a decrease in oxygen levels.

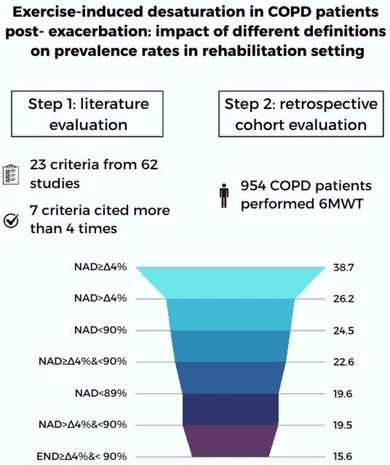

## 1 Introduction

The 6‐min walking test (6MWT) is widely utilized in clinical practice due to its ease of administration, minimal equipment requirements [1], and strong correlation with patient outcomes [2, 3]. It provides valuable insights into functional capacity, response to therapy, and prognosis in chronic cardiopulmonary conditions [4].

Several studies have demonstrated the reliability of measuring SpO_2_ during the 6MWT [5, 6], and a recent ERS/ATS [7] recommendation indicates it as the standard assessment of oxygen desaturation, incorporating continuous pulse oximetry to monitor SpO_2_ and heart rate in patients with chronic obstructive pulmonary disease (COPD).

COPD's progression involves deteriorating gas exchange in the lungs. Oxyhemoglobin desaturation typically occurs during exertion, even in subjects with normal gas exchange at rest [8].

Exercise‐induced oxygen desaturation (EID) can be highlighted in COPD during the 6MWT [9]. Patients with desaturation had a significantly higher mortality rate within 8 years than those without [10].

However, the prevalence of EID during the 6MWT varies widely [11, 12] due to the lack of a standardized definition, with different criteria used [13, 14, 15].

EID significantly influences clinical and functional outcomes [16, 17]; it marks functional decline in COPD [18] and is a key criterion for prescribing oxygen therapy during exertion [19]. Therefore, a consensus on standardized criteria is advisable.

The objectives of this study were to evaluate (a) different EID definitions in the literature, (b) the prevalence of EID during the 6MWT based on the most cited definitions in COPD patients admitted to pulmonary rehabilitation (PR), and (c) the agreement and concordance between different EID definitions.

## 2 Materials and Methods

### 2.1 Literature Evaluation

We searched MEDLINE and EMBASE databases from inception to February 15, 2025, using (“COPD” OR “chronic obstructive pulmonary disease”) AND (“oxygen” AND “desaturation”) AND “exercise”. We included all COPD patient studies reporting a desaturation criterion during the 6MWT, without age, ethnicity, or sex restrictions. Studies lacking desaturation measures or not in English were excluded. One investigator (S.B.) screened titles and abstracts by inclusion criteria, retrieving potentially eligible studies. Excluded studies and reasons were recorded. Data were entered into a Microsoft Excel database, independently checked by a second reviewer (C.S.). Eligibility disagreements were resolved by discussion, with a third reviewer (M.P.) available if needed. For each included study, the authors, publication year, and all EID definitions were recorded. Identical definitions were synthesized into criteria. For each criterion, we counted included studies and collected references.

### 2.2 EID Prevalence Estimation

To evaluate EID prevalence during the 6MWT by different definitions, we performed a retrospective cross‐sectional analysis of our institutional database containing data of consecutive COPD patients admitted to the ICS Maugeri PR network (rehabilitation units of nine centers: Ginosa, Bari, Pavia, Montescano, Tradate, Lumezzane, Veruno, Milano, Telese; Italy), which is a referral hospital for chronic disease diagnosis, care, and rehabilitation. Maugeri network PR units share common assessment, diagnostic, and management procedures [20]. COPD diagnosis was based on the administrative discharge diagnosis (DRG 88), a system classifying hospital cases by diagnoses and resource use. Physicians assigned the COPD discharge diagnosis using historical or measured spirometry per GOLD 2022 criteria. Patients were admitted from acute care or home via general practitioner referral. On admission, all participants were clinically stable (pH > 7.35 and SpO_2_ > 90% at rest) and received regular medical treatment per current guidelines for their disease stage [21].

We included all 6MWTs performed at the start of PR for COPD patients admitted, after exacerbation, between June 28, 2022, and June 19, 2024. The 6MWT was performed according to accepted standards [7]: Patients walked as far as possible on a flat corridor in 6 min, and the distance (6MWD) was recorded. Arterial oxygen saturation (SpO_2_), heart rate (HR), blood pressure, and dyspnea/fatigue (Borg scale) were recorded pretest and posttest. SpO_2_ and HR were also recorded every minute via finger probe (Nonin WristOx 3150/8500); the lowest SpO_2_ level reached during the test (nadir/NAD) was recorded. The most recent 6MWT data per patient were analyzed, excluding tests with oxygen supplementation.

The study, approved by the Ethics Committee (EC Lombardia 6 Prot. 18390/25; 31/03/2025), followed the Declaration of Helsinki.

We collected patient demographics (age, sex), anthropometrics (body mass index [BMI]), Cumulative Illness Rating Scale with its Comorbidity Complexity Index (CIRS) [22], Barthel index dyspnea (BiD) [23], COPD Assessment Test (CAT) [24], and Medical Research Council (MRC) dyspnea scale [25] for each 6MWT.

Following literature review, EID prevalence data during 6MWT were analyzed using frequently cited criteria.

We then calculated agreement and concordance between different criteria.

### 2.3 Statistical Analysis

Statistical analysis was performed using STATA 11.3 (StataCorp LLC) and Python (Python Software Foundation, USA). All results were presented with a 95% confidence interval. For descriptive statistics, continuous variables were analyzed using the mean and standard deviation, whereas dichotomous or categorical variables were presented as percentages. Parametric statistics were applied to numerical variables not solely based on the assumption of a Gaussian distribution (as assessed by the Shapiro–Wilk test) but also considering sample size, unimodality, and the symmetry of the variable distributions.

#### 2.3.1 Prevalence Analysis

To conduct this study, we decided to include definitions that were mentioned more than three times (i.e., at least four times) in the studies identified in our literature search. A priori, the sample size for the prevalence study was calculated based on a conservative estimate of the prevalence rate, set at 61.7% according to the literature [12]. A 95% confidence level (*z* = 1.96) and a 5% margin of error (*E* = 0.05) were used to determine the required sample size. Using the criterion for prevalence studies, the required sample size was estimated to be 364 participants.

#### 2.3.2 Agreement and Concordance

Fleiss' kappa was used to assess agreement between definitions, and Cohen's kappa was used for pairwise comparisons. In both analyses, agreement was interpreted as excellent for a coefficient above 0.80, good between 0.60 and 0.79, moderate between 0.40 and 0.59, and poor below 0.39 [26]. Additionally, we calculated the raw percentage of agreement to represent the proportion of cases in which both definitions identified desaturation in the same subjects. Cohen's kappa was used to complement this by providing a measure of agreement corrected for chance.

## 3 Results

**FIGURE 1:**
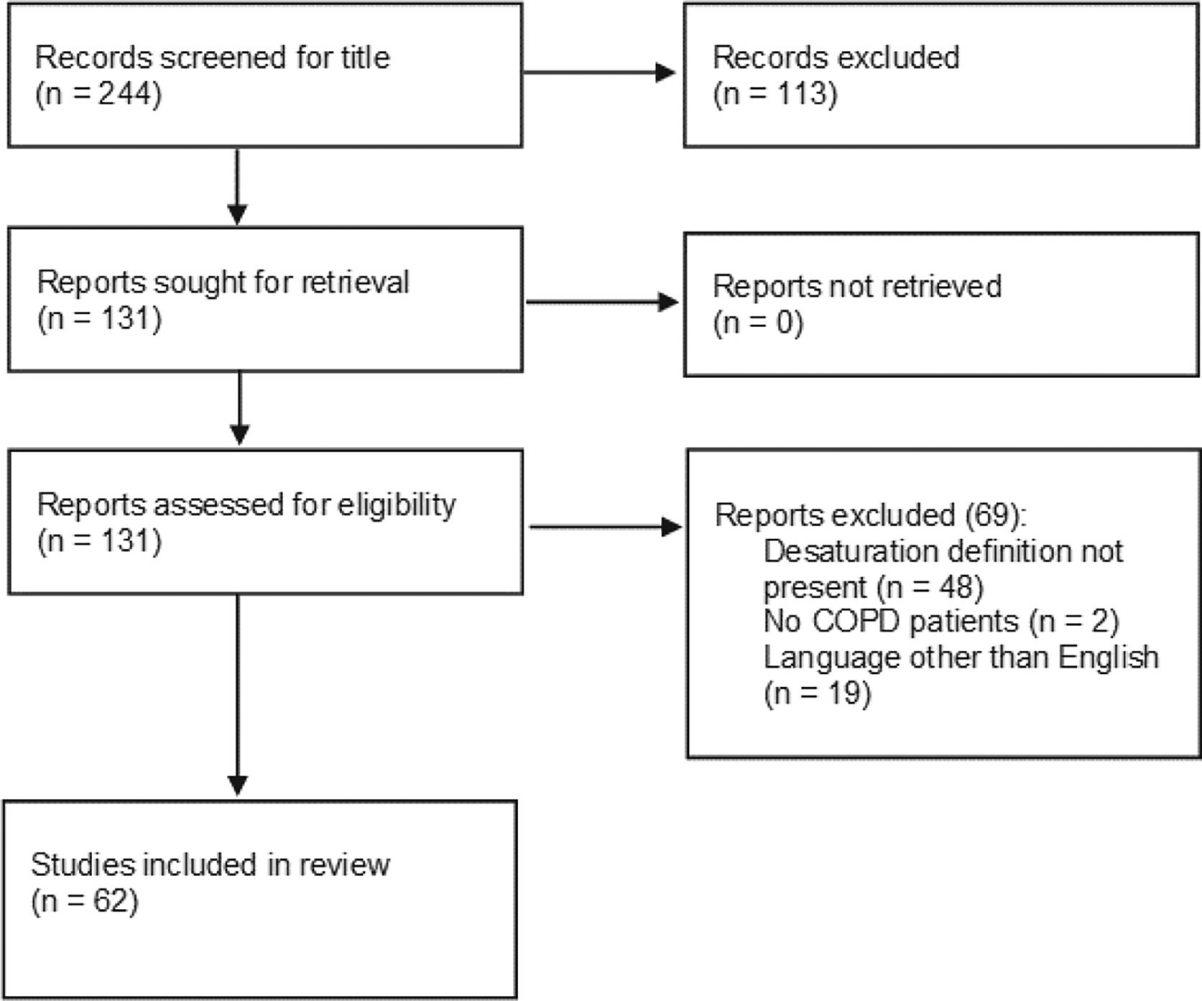
Flow chart of the literature searching. COPD = chronic obstructive pulmonary disease; *n* = number.

We identified 244 potentially relevant articles, of which 62 met the eligibility criteria for analysis of the definition of EID (the study profile is described in Figure 1, and the 62 articles are listed in the Supporting Information). A total of 113 studies were excluded on the basis of the title, as they were not relevant to the analysis conducted, whereas a further 69 were excluded after reviewing the abstract or full text, mainly because of the lack of a definition of EID during the 6MWT or because they were not in English. The search was conducted in February 2025. Table 1 shows all the definitions of EID during the 6MWT identified, the associated acronym for the selected definitions, the number of citations found for each criterion in the present review literature, and the references for each cited definition. The EID definitions (Table 1) are listed in descending order of citation. Definitions generally fall into three desaturation categories: those focusing on minimum saturation value (category: saturation‐based), those on saturation drop (category: nadir), and those combining both (category: combined). The most common description refers to the occurrence of a decrease in SpO_2_ of at least 4% during walking.

**TABLE 1:** Definition of exercise‐induced desaturation during the 6MWT evaluated in the literature.

Criterion	No. of citations	References in the Supporting Information, ^a^
A: ΔSpO_2_ from baseline to nadir ≥ 4%	19	1–19
B: SpO_2_ < 90%	17	2, 3, 6, 9, 13, 16, 17, 20–29
C: ΔSpO_2_ from baseline to nadir > 4% + SpO_2_ < 90%	9	11, 20, 22, 30–35
D: SpO_2_ ≤ 88%	8	3, 8–10, 36–39
E: ΔSpO_2_ from baseline to end test ≥ 4% + SpO_2_ end test < 90%	6	40–45
F: ΔSpO_2_ from baseline to nadir ≥ 4% + SpO_2_ < 90%	6	14, 30, 46–49
G: ΔSpO_2_ from baseline to nadir > 4%	4	20–22, 50
SpO_2_ < 88%	3	50–52
SpO_2_ end test ≤ 88%	3	3, 7, 53
ΔSpO_2_ from baseline to nadir ≥ 4% + SpO_2_ ≤ 88%	2	19, 54
ΔSpO_2_ from baseline to nadir ≥ 4% + SpO_2_ ≥ 90%	1	46
ΔSpO_2_ from baseline to nadir ≥ 4% + SpO_2_ < 88%	1	55
ΔSpO_2_ from baseline to nadir ≥ 4% for more than 1 min	1	56
ΔSpO_2_ from baseline to end test ≥ 4% + SpO_2_ end test ≤ 88%	1	7
SpO_2_ end test < 90%	1	12
ΔSpO_2_ from baseline to nadir > 5%	1	57
ΔSpO_2_ from baseline to nadir > 10%	1	58
Average SpO_2_ < 88%	1	59
ΔSpO_2_ > 4% for the last 3 min	1	14
ΔSpO_2_ ≥ 4% over 3 min	1	60
85% ≤ ΔSpO_2_ from baseline to nadir ≤ 90%	1	61
ΔSpO_2_ from baseline to nadir < 85%	1	61
ΔSpO_2_ ≥ 4% during at least the last 3 min	1	62

*Note:* A: ΔSpO_2_ from baseline to nadir ≥ 4% = NAD ≥ Δ4% (category: nadir); B: SpO_2_ < 90% = NAD < 90% (category: saturation‐based); C: ΔSpO_2_ from baseline to nadir > 4% + SpO_2_ < 90% = NAD > Δ4% and < 90% (category: combined); D: SpO_2_ ≤ 88% = NAD < 89% (category: saturation‐based); E: ΔSpO_2_ from baseline to end test ≥ 4% + SpO_2_ end test < 90% = END ≥ Δ4% and < 90% (category: combined); F: ΔSpO_2_ from baseline to nadir ≥ 4% + SpO_2_ < 90% = NAD ≥ Δ4% and < 90% (category: combined); G: ΔSpO_2_ from baseline to nadir > 4% = NAD > Δ4% (category: nadir).

Abbreviations: Δ = delta; END = SpO_2_ at 6MWT end; NAD = nadir SpO_2_; SpO_2_ = percentage of oxygen saturation.

^a^ See Supporting Information for more details about references.

To ensure the clinical and scientific relevance of the criteria analyzed, we adopted a selection threshold based on the citation distribution identified in our scoping review (mean citations: 3.33 ± 5.07). Consequently, only definitions cited at least four times were included in the prevalence analysis. This approach was chosen to focus on the most widely recognized criteria in the literature, avoiding the inclusion of highly specific or nonrecurring definitions that would limit the generalizability and statistical robustness of our comparative findings.

### 3.1 Study of Prevalence

Our electronic database contained data from 2334 6MWTs performed by patients with COPD on admission to the PR. Of these, 799 tests were excluded because they referred to patients with respiratory failure who performed the test with supplemental oxygen therapy, whereas 581 were excluded because the patients were unable to complete the test due to motor and mobility impairment or physical disability. In the end, we used a dataset of 954 6MWTs for this study.

Table 2 describes the available data on the anthropometric and clinical characteristics of the patients who performed the 6MWTs.

**TABLE 2:** Anthropometric and clinical characteristics of patients who performed the 6MWTs.

Measures	Analyzed patients ( *N* = 954)
Age, years	70.49 ± 10.59
BMI, kg/m ^2^	28.59 ± 7.11
CIRS, score ( *n* = 521)	3.89 ± 1.97
BiD, score	29.88 ± 15.95
MRC, score ( *n* = 715)	2.57 ± 1.13
CAT, score ( *n* = 619)	20.91 ± 6.84
6MWT, meters	311.76 ± 122.96
SpO_2_ at baseline, %	94.78 ± 2.0
HR at baseline, bpm	77.80 ± 14.27
Borg fatigue at baseline, score	0.44 ± 1.06
Borg dyspnea at baseline, score	0.59 ± 1.01
SpO_2_ at the end, %	92.62 ± 3.73
HR at the end, bpm	95.73 ± 16.00
Borg fatigue at the end, score	3.99 ± 2.55
Borg dyspnea at the end, score	5.17 ± 2.69

*Note:* Results are expressed as mean ± standard deviation.

Abbreviations: 6MWT, 6‐min walking test; BiD, Barthel index dyspnea; BMI, body mass index; CAT, COPD Assessment Test; CIRS, Cumulative Illness Rating Scale_Comorbidity Complexity Index; HR, heart rate; MRC, Medical Research Council.

The mean CIRS score indicates a moderate level of comorbidity among the patients. They had moderate to severe dyspnea during activities of daily living, as indicated by the Barthel Index Dyspnea Score and the MRC scale, whereas the CAT score describes a moderate impact of the disease on patients' lives.

**FIGURE 2:**
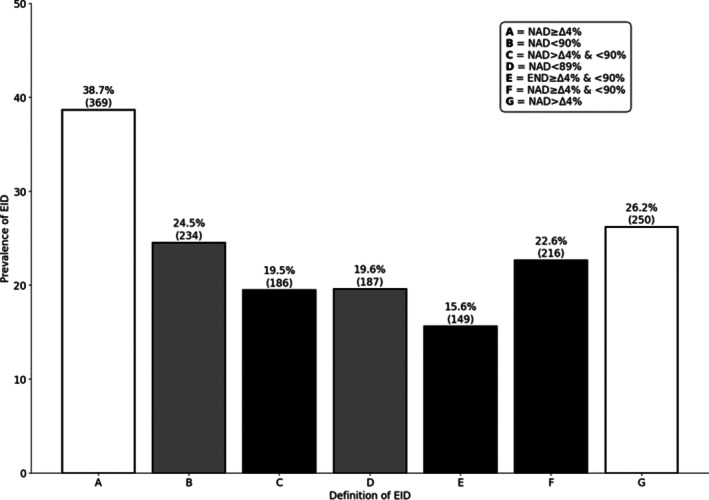
Prevalence of exercise‐induced desaturation according to different criteria. EID = exercise‐induced desaturation, NAD = nadir; NAD ≥ Δ4% = ΔSpO2 from baseline to nadir ≥ 4%;  NAD < 90% = SpO2 < 90%; NAD > Δ4% and < 90% = ΔSpO2 from baseline to nadir > 4% + SpO2 < 90%; NAD < 89% = SpO2 ≤ 88%; END ≥ Δ4% and < 90% = ΔSpO2 from baseline to end test ≥ 4% + SpO2 end test < 90%; NAD ≥ Δ4% and < 90% = ΔSpO2 from baseline to nadir ≥ 4% + SpO2 < 90%; NAD > Δ4% = ΔSpO2 from baseline to nadir > 4%; EID definitions: based on nadir (white), saturation‐based (grey), and combined (black).

The prevalence of oxygen desaturation among patients ranged from 15.6% to 38.7% according to the seven criteria chosen (Figure 2). Focusing on the definition that uses minimum oxygen saturation as the criterion to define EID (Criteria B and D), 24.5% of patients exhibited a saturation level below 90%, with approximately 20% having a saturation of 88% or lower.

However, the most consistent classifications were observed when looking at definitions based on the change in SpO_2_ levels (Criteria A and G). This approach identified 38.7% of patients who experienced a desaturation of at least 4% points during exercise, whereas 26.2% had a desaturation of more than 4% points.

Using the definition combining both the nadir values below 90% and the drop in saturation during the test (Criteria C and F), 22.6% of patients experienced oxygen desaturation with a drop of at least 4% and 19.5% with a drop of more than 4%. The smallest group, consisting of 15.6% of patients, was identified when the analysis was restricted to those who experienced both a drop of 4% or more from baseline to the end of the test and a low end‐test saturation.

To validate the cohort selection, a sensitivity analysis was conducted in a subgroup of 83 patients with available spirometric data (FEV1: 53.13% ± 22.84% prd; FVC: 74.72% ± 25.23% prd; FEV1/FVC: 53.31% ± 11.97% prd). The prevalence rates for EID within this confirmed subgroup were consistent with those observed in the total population; specifically, the proportion of patients identified with EID ranged from 19.3% (Criterion E) to 49.4% (Criterion A) across the seven analyzed criteria.

### 3.2 Agreement and Concordance Among EID Definitions

**FIGURE 3:**
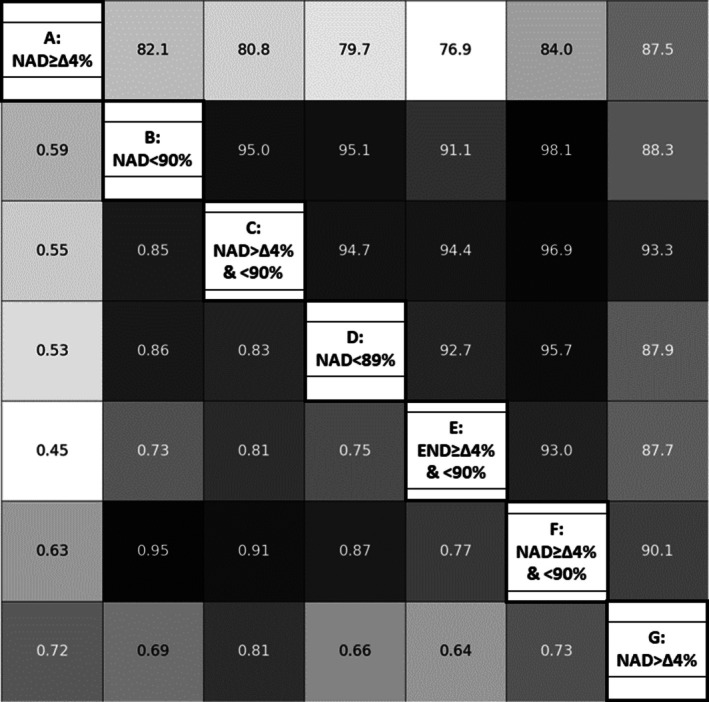
Heatmap of the one‐to‐one agreement (Cohen's kappa between pairs of EID definitions, boxes on the left of the diagonal) and concordance (percentage of concordant classifications between pairs of EID definitions, boxes on the right of the diagonal) between all EID definitions. EID = exercise‐induced desaturation, NAD = nadir. The values are color‐coded, with darker shades indicating higher values and lighter shades indicating lower values.

The heat map in Figure 3 describes the agreement and concordance between each definition of desaturation. As shown in Figure 3, the criterion NAD ≥ 4% (Criterion A) (considering the difference between basal SpO_2_ and nadir of 4% or more) shows low concordance with the other definitions. This indicates limited consistency in classification, suggesting that this criterion identifies patients with EID differently from the other methods. On the other hand, if we consider NAD < 90% (Criterion B) (as a nadir below 90% during the 6MWT), we see higher values of agreement, showing a strong agreement with the other classification criterion, especially with the criterion that combines the nadir below 90% and a fall in SpO_2_. This suggests that it consistently identifies patients who have had an EID, as do most of the other criteria.

Focusing on these two criteria alone, comparing the EID definition criteria with a nadir of < 90% (B: NAD < 90%) with the EID criteria with a nadir of < 90% and a delta between baseline and nadir of > 4 points (C: NAD > Δ4% and < 90%), results in an additional 48 patients being classified as having EID, highlighting the limited additional impact of the second criterion (Figure 2).

The overall agreement across the seven definitions was good, with a Fleiss' kappa for multiple agreements of 0.718.

## 4 Discussion

Our analysis of a large COPD population admitted to an inpatient PR program showed important differences in the prevalence of oxygen desaturation according to different definitions proposed in the recent literature.

The most common description refers to the occurrence of at least a 4% reduction of SpO_2_ during walking.

Good agreement and concordance were found between all EID definitions.

The majority of desaturation definitions use a drop cut‐off equal to or greater than 4%: The rationale is that most pulse oximeter manufacturers claim an accuracy of 2%, which is the standard deviation of the differences between the SpO_2_ by pulse oximetry and the SatO_2_ by co‐oximetry in extracted blood, associated with an expected error of 4% (two SDs) [27].

However, Milner et al. investigated the accuracy of pulse oximeters and found that a significant proportion of pulse oximeter sensors may be inaccurate, suggesting caution when using these devices alone to assess oxygen supplementation [28].

The definition that only considers a fall in SpO_2_ ≥ 4% (the less conservative) shows the highest number of patients with desaturation compared to all other definitions. However, this criterion does not take into account the GOLD guidelines, which refer to a minimum value of 90% to define a patient as hypoxic.

According to the GOLD guidelines [29] and in other studies [30], hypoxemia is diagnosed when pulse oximetry oxygen saturation is < 90%. Different authors used this value as a cut‐off to define the EID; some considered a nadir below 90% during all the tests, whereas others only evaluated the value at the end of the test. However, the literature indicates that focusing only on end‐exercise SpO_2_ may fail to capture transient desaturation that occurs during the test (better captured by mean or nadir SpO_2_) but subsequently recovers by the end.

Other authors have chosen to use both definitions: a drop in the saturation during the test and a saturation below 90%. Monitoring saturation throughout the entire test may, in fact, be more conservative and accurate to ensure that all patients falling below 90% saturation are properly defined.

We analyzed the agreement/concordance among different criteria to better understand how consistently different literature criteria classify individuals with an EID. Although the overall agreement between the seven definitions was found to be good, the criterion considering the difference between basal SpO_2_ and nadir of at least 4% shows low concordance with the other definitions, suggesting discrepancies in desaturation classification.

We have shown that the different criteria are not interchangeable because of the wide limits of agreement between them and due to a wide variability of patients classified as desaturators according to the different definitions.

Oxygen desaturation during the 6MWT in patients with COPD is a significant prognostic marker for mortality [10, 17], exacerbations [15], and decline in lung function [17], whereas the role of oxygen supplementation during exertion remains debated, although continuous oxygen supplementation appears to be associated with potential long‐term benefits [31].

Current criteria for initiating oxygen therapy are based on a resting PaO_2_ < 55 mmHg or a resting SpO_2_ < 88%, with oxygen supplementation given to bring the saturation to 90% [32]. However, guidelines do not indicate what to do in the case of only exertional desaturation. Uncertainty remains about the most appropriate time modality and criterion for starting oxygen supplementation under effort, implying a difficult decision for the clinician. The evidence supporting oxygen therapy for EID is contradictory, highlighting the need for more definitive research. In this context, the identification of patients who experience a desaturation of at least 4% during exertion, while maintaining an SpO_2_ above 90%, or the timing of desaturation during the test, could represent precursor elements for the subsequent development of overt EID. Thus, the number of patients who meet the criteria for ambulatory oxygen under effort varies according to the different criteria used. Considering the presence of EID in those patients with a desaturation of 4% or more to a value lower than 90%, Lewko [33] compared the incremental shuttle walk test (ISWT) and the 6MWT in COPD patients and found a large variation in EID depending on the test used. Given the debated results regarding mortality, performance, and dyspnea, it is still unclear what the best approach to oxygen supplementation during exertion is. Further studies are needed to confirm this hypothesis.

The evidence supporting oxygen supplementation in EID remains contradictory.

On the one hand, it has been shown that oxygen supplementation during the 6MWT can increase walk distance [34, 35] and reduce dyspnea in COPD patients who desaturate during the test [34, 35] and that those who had a better walk distance outcome with O_2_ also had a significantly lower mortality rate compared with nonresponders [36].

On the other hand, the addition of ambulatory supplemental oxygen does not appear to be more effective than placebo in the long term [37], and long‐term supplemental oxygen in patients with stable COPD and moderate desaturation at rest or during exercise did not benefit time to death or first hospitalization [13]. In a clinical context where the benefits of oxygen therapy for EID are still debated, it could be hypothesized that adopting a more conservative definition might help clinicians identify patients with more pronounced desaturation, potentially reducing the risk of over‐prescription. All these considerations reinforce the purpose of the present study, which, by highlighting significant differences in the definitions of EID, underlines the urgent need to standardize the whole issue. The use of different definitions with significant differences in EID rates could have organizational implications and costs for different healthcare systems.

Moreover, the lack of a standardized and shared definition of EID represents a major barrier to clinical research; without it, establishing a meaningful prognostic follow‐up or conducting prospective studies with comparable cohorts remains inherently flawed. Rather than assessing prognosis, the present study aims to highlight that this diagnostic fragmentation itself prevents a reliable assessment of long‐term clinical outcomes and may lead to the risk of over‐prescription, increased healthcare costs, and unnecessary patient burden.

Furthermore, this lack of standardization has direct implications for PR protocols and LTOT prescription during exertion. Although our study does not include follow‐up data on training responses and on long‐term outcomes (relapses, hospitalizations, survival), highlighting the variability in EID prevalence represents a necessary step toward aligning clinical practice with more effective and individualized treatment strategies.

The main limitation of our study is related to its retrospective cross‐sectional design, which only allows us to describe the impact of the different definitions tested on the prevalence of oxygen desaturation and precludes us from evaluating their prognostic relevance.

Furthermore, the absence of comprehensive clinical data—specifically lung function tests, arterial blood gas analysis, and DLCO—limits our ability to precisely stratify COPD severity and characterize the physiological impairment of our cohort; moreover, the absence of DLCO prevents us from analyzing the physiological correlation between different EID definitions and the underlying pulmonary pathology. Consequently, our findings rely on administrative diagnosis, and although they offer a meaningful snapshot of current clinical practice, future prospective studies incorporating detailed functional metrics are essential to validate these associations and further explore their clinical implications.

The use of DRG codes for patient identification is a known limitation; however, our sensitivity analysis conducted in a spirometry‐confirmed subgroup showed no significant differences in EID prevalence compared with the main cohort. This finding suggests that, within the specific context of a PR unit, the risk of misclassification bias is likely minimal.

Another limitation of our study is the limited generalizability of the findings, as our sample consisted of COPD patients admitted to PR following an exacerbation, representing a more severe and symptomatic subgroup than the overall COPD population.

On the other hand, we were able to obtain data from a large, unselected population of adults with COPD who were referred to our unit to evaluate the indication to start oxygen therapy during exertion, which is exactly the clinical context in which the definitions studied should be applied. Oxygen saturation seems to correlate with diffusing capacity, FEV_1_/FVC, and peak expiratory flow, but this pulmonary function does not have a positive predictive value in determining exertional desaturation [38]. Unfortunately, this study is not able to add any information on this point. Therefore, the reasons why patients with COPD and mild resting hypoxemia develop exertional desaturation remain unknown.

In conclusion, our analysis of a large COPD population during an inpatient PR program reveals a significant difference in the prevalence of oxygen desaturation based on different definitions. The most common criterion is a ≥ 4% decrease in SpO_2_ during walking. Although there is good agreement between definitions, the most conservative approach considers both the drop in SpO_2_ and the SpO_2_ at the end of the test, identifying desaturation in 15% of patients. A uniform definition of exertional desaturation to describe a decrease in oxygen levels in people with COPD remains mandatory. Future studies will be necessary to determine which definition is most appropriate, predictive for outcomes, and useful for prescribing oxygen under effort.

## Author Contributions

All authors participated actively in the study, giving substantial contributions and approving the final version to be published. B.S.: conceptualization, methodology, investigation, data curation, formal analysis, visualization, writing – original draft, writing – review and editing. M.V.: conceptualization, methodology, supervision, writing – original draft, writing – review and editing. M.P.: methodology, formal analysis, writing – review and editing. C.S.: investigation, data curation, writing – review and editing. R.M.: investigation, writing – review and editing. F.D.N.: investigation, writing – review and editing.

## Funding

This work was supported by the “Ricerca Corrente” funding scheme of the Ministry of Health, Italy.

## Ethics Statement

Ethical approval was obtained for this study (EC Lombardia 6 Prot. 18390/25; 31/03/2025).

## Conflicts of Interest

The authors declare no conflicts of interest.

## Supporting information


**Data S1:** List of 62 articles meeting the eligibility criteria for the analysis of the EID definition.

## Data Availability

The data that support the findings of this study are available from the corresponding author upon reasonable request.
