# Supplementary material for: Exercise‐Induced Desaturation in COPD Patients Postexacerbation: Impact of Different Definitions on Prevalence Rates in a Rehabilitation Setting
Source: Clin Respir J. 2026 May 31;20(6):e70199. doi: 10.1111/crj.70199 (PMC13240415; doi:10.1111/crj.70199)
Supplement: Supplementary file 1 — Data S1: List of 62 articles meeting the eligibility criteria for the analysis of the EID definition. [file CRJ-20-e70199-s001.docx]

**Exercise-induced desaturation in COPD patients post-exacerbation: impact of different definitions on prevalence rates in a rehabilitation setting**

**Supplementary material**

**List of 62 articles meeting the eligibility criteria for the analysis of the EID definition**

1. Batista KS, Cézar ID, Benedetto IG, C da Silva RM, Wagner LE, Pereira da Silva D, Sanches PR, Gazzana MB, Knorst MM, de-Torres JP, Neder JA, Berton DC. Continuous Monitoring of Pulse Oximetry During the 6-Minute Walk Test Improves Clinical Outcomes Prediction in COPD. Respir Care. 2023 Jan;68(1):92-100. doi: 10.4187/respcare.10091.
2. Lee KY, Wu SM, Kou HY, Chen KY, Chuang HC, Feng PH, Chung KF, Ito K, Chen TT, Sun WL, Liu WT, Tseng CH, Ho SC. Association of air pollution exposure with exercise-induced oxygen desaturation in COPD. Respir Res. 2022 Mar 31;23(1):77. doi: 10.1186/s12931-022-02000-1.
3. Kim C, Ko Y, Lee JS, Rhee CK, Lee JH, Moon JY, Lim SY, Yoo KH, Seo JB, Oh YM, Lee SD, Park YB. Predicting long-term mortality with two different criteria of exercise-induced desaturation in COPD. Respir Med. 2021 Jun;182:106393. doi: 10.1016/j.rmed.2021.106393. Epub 2021 Apr 6.
4. Fernandes AL, Neves I, Luís G, Camilo Z, Cabrita B, Dias S, Ferreira J, Simão P. Is the 1-Minute Sit-To-Stand Test a Good Tool to Evaluate Exertional Oxygen Desaturation in Chronic Obstructive Pulmonary Disease? Diagnostics (Basel). 2021 Jan 22;11(2):159. doi: 10.3390/diagnostics11020159.
5. Vieira EB, Degani-Costa LH, Amorim BC, Oliveira LB, Miranda-Silva T, Sperandio PC, Medeiros WM, Arbex FF, Ramos RP, Nery LE. Modified BODE Index to Predict Mortality in Individuals With COPD: The Role of 4-Min Step Test. Respir Care. 2020 Jul;65(7):977-983. doi: 10.4187/respcare.06991. Epub 2020 Jan 28.
6. Misu S, Kaneko M, Sakai H, Oki Y, Fujimoto Y, Ishikawa A, Ono R. Exercise-Induced Oxygen Desaturation as a Predictive Factor for Longitudinal Decline in 6-Minute Walk Distance in Subjects With COPD. Respir Care. 2019 Feb;64(2):145-152. doi: 10.4187/respcare.06169. Epub 2018 Oct 16.
7. Sadaka AS, Montgomery AJ, Mourad SM, Polkey MI, Hopkinson NS. Exercise response to oxygen supplementation is not associated with survival in hypoxemic patients with obstructive lung disease. Int J Chron Obstruct Pulmon Dis. 2018 May 17;13:1607-1612. doi: 10.2147/COPD.S163119.
8. Ergan B, Nava S. Long-Term Oxygen Therapy in COPD Patients Who Do Not Meet the Actual Recommendations. COPD. 2017 Jun;14(3):351-366. doi: 10.1080/15412555.2017.1319918. Epub 2017 May 16.
9. Oki Y, Kaneko M, Fujimoto Y, Sakai H, Misu S, Mitani Y, Yamaguchi T, Yasuda H, Ishikawa A. Usefulness of the 6-minute walk test as a screening test for pulmonary arterial enlargement in COPD. Int J Chron Obstruct Pulmon Dis. 2016 Nov 22;11:2869-2875. doi: 10.2147/COPD.S114497.
10. Dogra AC, Gupta U, Sarkar M, Padam A. Exercise-induced desaturation in patients with chronic obstructive pulmonary disease on six-minute walk test. Lung India. 2015 Jul-Aug;32(4):320-5. doi: 10.4103/0970-2113.159550.
11. García-Talavera I, Jiménez González P, Dorta Sánchez R. Exercise-induced oxygen desaturation in chronic obstructive pulmonary disease patients. Arch Bronconeumol. 2015 Oct;51(10):481-2. English, Spanish. doi: 10.1016/j.arbres.2015.01.004. Epub 2015 Mar 29.
12. Kim C, Seo JB, Lee SM, Lee JS, Huh JW, Lee JH, Ra SW, Lee JH, Kim EK, Kim TH, Kim WJ, Lee SM, Lee SY, Lim SY, Shin TR, Yoon HI, Sheen SS, Oh YM, Park YB, Lee SD. Exertional desaturation as a predictor of rapid lung function decline in COPD. Respiration. 2013;86(2):109-16. doi: 10.1159/000342891. Epub 2012 Dec 11.
13. Fiore C, Lee A, McDonald C, Hill C, Holland A. Should oxyhaemoglobin saturation be monitored continuously during the 6-minute walk test? Chron Respir Dis. 2011;8(3):181-4. doi: 10.1177/1479972311407355.
14. Panos RJ, Eschenbacher W. Exertional desaturation in patients with chronic obstructive pulmonary disease. COPD. 2009 Dec;6(6):478-87. doi: 10.3109/15412550903341497.
15. Chatterjee AB, Rissmiller RW, Meade K, Paladenech C, Conforti J, Adair NE, Haponik EF, Chin R Jr. Reproducibility of the 6-minute walk test for ambulatory oxygen prescription. Respiration. 2010;79(2):121-7. doi: 10.1159/000220343. Epub 2009 May 20.
16. Salzman SH. The 6-min walk test: clinical and research role, technique, coding, and reimbursement. Chest. 2009 May;135(5):1345-1352. doi: 10.1378/chest.07-1682.
17. Casanova C, Cote C, Marin JM, Pinto-Plata V, de Torres JP, Aguirre-Jaíme A, Vassaux C, Celli BR. Distance and oxygen desaturation during the 6-min walk test as predictors of long-term mortality in patients with COPD. Chest. 2008 Oct;134(4):746-752. doi: 10.1378/chest.08-0520. Epub 2008 Jul 14.
18. Hadeli KO, Siegel EM, Sherrill DL, Beck KC, Enright PL. Predictors of oxygen desaturation during submaximal exercise in 8,000 patients. Chest. 2001 Jul;120(1):88-92. doi: 10.1378/chest.120.1.88.
19. Knower MT, Dunagan DP, Adair NE, Chin R Jr. Baseline oxygen saturation predicts exercise desaturation below prescription threshold in patients with chronic obstructive pulmonary disease. Arch Intern Med. 2001 Mar 12;161(5):732-6. doi: 10.1001/archinte.161.5.732.
20. Wu YF, Shu X, Wang S, Xu X, Sun PL. Accurate identification of oxygen desaturation status in COPD by using classifier ensemble. PLoS One. 2025 Feb 5;20(2):e0318837. doi: 10.1371/journal.pone.0318837.
21. Tsai ML, Li CL, Chang HC, Tsai YC, Tseng CW, Liu SF. The Relationship between Exertional Desaturation and Pulmonary Function, Exercise Capacity, or Medical Costs in Chronic Obstructive Pulmonary Disease Patients. Medicina (Kaunas). 2023 Feb 17;59(2):391. doi: 10.3390/medicina59020391.
22. Perez T, Deslée G, Burgel PR, Caillaud D, Le Rouzic O, Zysman M, Escamilla R, Jebrak G, Chanez P, Court-Fortune I, Brinchault-Rabin G, Nesme-Meyer P, Paillasseur JL, Roche N; Initiatives BPCO Scientific Committee. Predictors in routine practice of 6-min walking distance and oxygen desaturation in patients with COPD: impact of comorbidities. Int J Chron Obstruct Pulmon Dis. 2019 Jul 2;14:1399-1410. doi: 10.2147/COPD.S188412.
23. Narewski ER, Blackford AL, Lammi MR, Fuhlbrigge AL, Soler X, Albert R, Criner GJ; Long-Term Oxygen Treatment Trial Research Group. Clinical Differences in COPD Patients with Variable Patterns of Hypoxemia. Chronic Obstr Pulm Dis. 2018 Apr 28;5(3):167-176. doi: 10.15326/jcopdf.5.3.2017.0175.
24. Lacasse Y, Tan AM, Maltais F, Krishnan JA. Home Oxygen in Chronic Obstructive Pulmonary Disease. Am J Respir Crit Care Med. 2018 May 15;197(10):1254-1264. doi: 10.1164/rccm.201802-0382CI.
25. Magnet FS, Storre JH, Windisch W. Home oxygen therapy: evidence versus reality. Expert Rev Respir Med. 2017 Jun;11(6):425-441. doi: 10.1080/17476348.2017.1325323. Epub 2017 May 10.
26. Furlan L, Erba L; GrAM (Gruppo di Autoformazione Metodologica). Long-term oxygen for COPD with moderate desaturation. Intern Emerg Med. 2017 Mar;12(2):239-240. doi: 10.1007/s11739-017-1616-x. Epub 2017 Feb 10.
27. Long-Term Oxygen Treatment Trial Research Group; Albert RK, Au DH, Blackford AL, Casaburi R, Cooper JA Jr, Criner GJ, Diaz P, Fuhlbrigge AL, Gay SE, Kanner RE, MacIntyre N, Martinez FJ, Panos RJ, Piantadosi S, Sciurba F, Shade D, Stibolt T, Stoller JK, Wise R, Yusen RD, Tonascia J, Sternberg AL, Bailey W. A Randomized Trial of Long-Term Oxygen for COPD with Moderate Desaturation. N Engl J Med. 2016 Oct 27;375(17):1617-1627. doi: 10.1056/NEJMoa1604344.
28. Alison JA, McKeough ZJ, Jenkins SC, Holland AE, Hill K, Morris NR, Leung RW, Williamson KA, Spencer LM, Hill CJ, Lee AL, Seale H, Cecins N, McDonald CF. A randomised controlled trial of supplemental oxygen versus medical air during exercise training in people with chronic obstructive pulmonary disease: supplemental oxygen in pulmonary rehabilitation trial (SuppORT) (Protocol). BMC Pulm Med. 2016 Feb 4;16:25. doi: 10.1186/s12890-016-0186-4.
29. Stoller JK, Aboussouan LS, Kanner RE, Wilson LA, Diaz P, Wise R; LOTT Research Group. Characteristics of Alpha-1 Antitrypsin-Deficient Individuals in the Long-term Oxygen Treatment Trial and Comparison with Other Subjects with Chronic Obstructive Pulmonary Disease. Ann Am Thorac Soc. 2015 Dec;12(12):1796-804. doi: 10.1513/AnnalsATS.201506-389OC.
30. McKeough Z, Leung R, Neo JH, Jenkins S, Holland A, Hill K, Morris N, Spencer L, Hill C, Lee A, Seale H, Cecins N, McDonald C, Alison J. Shuttle walk tests in people with COPD who demonstrate exercise-induced oxygen desaturation: An analysis of test repeatability and cardiorespiratory responses. Chron Respir Dis. 2018 May;15(2):131-137. doi: 10.1177/1479972317729051. Epub 2017 Aug 30.
31. Wilson S, Cecins N, Jenkins S, Melang M, Singh B, Hill K. Comparing finger and forehead sensors to measure oxygen saturation in people with chronic obstructive pulmonary disease. Respirology. 2013 Oct;18(7):1143-7. doi: 10.1111/resp.12129.
32. Crisafulli E, Iattoni A, Venturelli E, Siscaro G, Beneventi C, Cesario A, Clini EM. Predicting walking-induced oxygen desaturations in COPD patients: a statistical model. Respir Care. 2013 Sep;58(9):1495-503. doi: 10.4187/respcare.02321. Epub 2013 Jan 29.
33. van Gestel AJ, Clarenbach CF, Stöwhas AC, Teschler S, Russi EW, Teschler H, Kohler M. Prevalence and prediction of exercise-induced oxygen desaturation in patients with chronic obstructive pulmonary disease. Respiration. 2012;84(5):353-9. doi: 10.1159/000332833. Epub 2012 Jan 20.
34. Sandland CJ, Morgan MDL, Singh SJ. Patterns of domestic activity and ambulatory oxygen usage in COPD. Chest. 2008 Oct;134(4):753-760. doi: 10.1378/chest.07-1848. Epub 2008 Jul 14.
35. Jones AY, Yu WC, Mok NS, Yeung OY, Cheng HC, Dean E. Exercise-induced desaturation and electrocardiogram changes in people with severe lung disease: an exploratory investigation of 25 serial cases. Heart Lung. 2006 Nov-Dec;35(6):397-404. doi: 10.1016/j.hrtlng.2006.05.001.
36. Wang CH, Lo CY, Huang HY, Wang TY, Weng CM, Chen CJ, Huang YC, Chung FT, Lin CW, Chung KF, Kuo HP. Oxygen Desaturation Is Associated With Fibrocyte Activation via Epidermal Growth Factor Receptor/Hypoxia-Inducible Factor-1α Axis in Chronic Obstructive Pulmonary Disease. Front Immunol. 2022 May 12;13:852713. doi: 10.3389/fimmu.2022.852713.
37. Giovacchini CX, Mathews AM, Lawlor BR, MacIntyre NR. Titrating Oxygen Requirements During Exercise: Evaluation of a Standardized Single Walk Test Protocol. Chest. 2018 Apr;153(4):922-928. doi: 10.1016/j.chest.2017.11.009. Epub 2017 Nov 21.
38. Andrianopoulos V, Wouters EF, Pinto-Plata VM, Vanfleteren LE, Bakke PS, Franssen FM, Agusti A, MacNee W, Rennard SI, Tal-Singer R, Vogiatzis I, Vestbo J, Celli BR, Spruit MA. Prognostic value of variables derived from the six-minute walk test in patients with COPD: Results from the ECLIPSE study. Respir Med. 2015 Sep;109(9):1138-46. doi: 10.1016/j.rmed.2015.06.013. Epub 2015 Jun 25.
39. Andrianopoulos V, Franssen FM, Peeters JP, Ubachs TJ, Bukari H, Groenen M, Burtin C, Vogiatzis I, Wouters EF, Spruit MA. Exercise-induced oxygen desaturation in COPD patients without resting hypoxemia. Respir Physiol Neurobiol. 2014 Jan 1;190:40-6. doi: 10.1016/j.resp.2013.10.002. Epub 2013 Oct 9.
40. Chiu LC, Hsu PC, Yen TH, Kuo SC, Fang YF, Lo YL, Lin SM, Yang CT, Lee CS. Blood Cadmium Levels and Oxygen Desaturation during the 6-Minute Walk Test in Patients with Chronic Obstructive Pulmonary Disease. Medicina (Kaunas). 2021 Oct 25;57(11):1160. doi: 10.3390/medicina57111160.
41. Waatevik M, Frisk B, Real FG, Hardie JA, Bakke P, Eagan TM, Johannessen A. CT-defined emphysema in COPD patients and risk for change in desaturation status in 6-min walk test. Respir Med. 2021 Oct;187:106542. doi: 10.1016/j.rmed.2021.106542. Epub 2021 Jul 23.
42. Chang CH, Lin HC, Yang CH, Gan ST, Huang CH, Chung FT, Hu HC, Lin SM, Chang CH. Factors Associated with Exercise-Induced Desaturation in Patients with Chronic Obstructive Pulmonary Disease. Int J Chron Obstruct Pulmon Dis. 2020 Oct 23;15:2643-2652. doi: 10.2147/COPD.S272511.
43. Waatevik M, Johannessen A, Gomez Real F, Aanerud M, Hardie JA, Bakke PS, Lind Eagan TM. Oxygen desaturation in 6-min walk test is a risk factor for adverse outcomes in COPD. Eur Respir J. 2016 Jul;48(1):82-91. doi: 10.1183/13993003.00975-2015. Epub 2016 Apr 13.
44. Rodrigues MK, Oliveira MF, Soares A, Treptow E, Neder JA. Additive effects of non-invasive ventilation to hyperoxia on cerebral oxygenation in COPD patients with exercise-related O2 desaturation. Clin Physiol Funct Imaging. 2013 Jul;33(4):274-81. doi: 10.1111/cpf.12024. Epub 2013 Jan 21.
45. Waatevik M, Johannessen A, Hardie JA, Bjordal JM, Aukrust P, Bakke PS, Eagan TM. Different COPD disease characteristics are related to different outcomes in the 6-minute walk test. COPD. 2012 Jun;9(3):227-34. doi: 10.3109/15412555.2011.650240. Epub 2012 Apr 12.
46. Weisenburger G, Bunel V, Godet C, Salpin M, Mouren D, Menonville CT, Goletto T, Marceau A, Borie R, Debray MP, Mal H. An underrecognized phenotype of pulmonary emphysema with marked pulmonary gas exchange but with mild or moderate airway obstruction. Respir Med Res. 2024 Nov;86:101086. doi: 10.1016/j.resmer.2024.101086. Epub 2024 Jul 27.
47. Vitacca M, Paneroni M, Braghiroli A, Balbi B, Aliani M, Guido P, Fanfulla F, Pertosa M, Ceriana P, Zampogna E, Raccanelli R, Sarno N, Spanevello A, Maniscalco M, Malovini A, Ambrosino N. Exercise capacity and comorbidities in patients with obstructive sleep apnea. J Clin Sleep Med. 2020 Apr 15;16(4):531-538. doi: 10.5664/jcsm.8258.
48. Roberts MM, Cho JG, Sandoz JS, Wheatley JR. Oxygen desaturation and adverse events during 6-min walk testing in patients with COPD. Respirology. 2015 Apr;20(3):419-25. doi: 10.1111/resp.12471. Epub 2015 Jan 20.
49. Jenkins S, Čečins N. Six-minute walk test: observed adverse events and oxygen desaturation in a large cohort of patients with chronic lung disease. Intern Med J. 2011 May;41(5):416-22. doi: 10.1111/j.1445-5994.2010.02169.x. Epub 2010 Jan 4.
50. Kawachi S, Yamamoto S, Nishie K, Yamaga T, Shibuya M, Sakai Y, Fujimoto K. The effectiveness of supplemental oxygen during exercise training in patients with chronic obstructive pulmonary disease who show severe exercise-induced desaturation: a protocol for a meta-regression analysis and systematic review. Syst Rev. 2021 Apr 14;10(1):110. doi: 10.1186/s13643-021-01667-9.
51. Agarwala P, Salzman SH. Six-Minute Walk Test: Clinical Role, Technique, Coding, and Reimbursement. Chest. 2020 Mar;157(3):603-611. doi: 10.1016/j.chest.2019.10.014. Epub 2019 Nov 2.
52. Ejiofor SI, Bayliss S, Gassamma A, Turner AM. Ambulatory Oxygen for Exercise-Induced Desaturation and Dyspnea in Chronic Obstructive Pulmonary Disease (COPD): Systematic Review and Meta-Analysis. Chronic Obstr Pulm Dis. 2016 Jan 6;3(1):419-434. doi: 10.15326/jcopdf.3.1.2015.0146.
53. Andrianopoulos V, Celli BR, Franssen FM, Pinto-Plata VM, Calverley PM, Vanfleteren LE, Vogiatzis I, Vestbo J, Agusti A, Bakke PS, Rennard SI, MacNee W, Tal-Singer R, Yates JC, Wouters EF, Spruit MA. Determinants of exercise-induced oxygen desaturation including pulmonary emphysema in COPD: Results from the ECLIPSE study. Respir Med. 2016 Oct;119:87-95. doi: 10.1016/j.rmed.2016.08.023. Epub 2016 Aug 25.
54. Kalinov RI, Marinov BI, Stoyanova DI, Hodgev VA, Vladimirova-Kitova LG, Nikolov FP, Kostianev SS. Desaturation during Physical Exercise in COPD Patients - a Stable-over-time Phenomenon. Folia Med (Plovdiv). 2019 Jun 1;61(2):204-212. doi: 10.2478/folmed-2018-0079.
55. García-Talavera I, Figueira-Gonçalves JM, Golpe R, Esteban C, Amado C, Pérez-Méndez LI, Aramburu A, Conde-Martel A. Early Desaturation During 6-Minute Walk Test is a Predictor of Mortality in COPD. Lung. 2023 Apr;201(2):217-224. doi: 10.1007/s00408-023-00613-x. Epub 2023 Apr 10.
56. Zafar MA, Tsuang W, Lach L, Eschenbacher W, Panos RJ. Dynamic hyperinflation correlates with exertional oxygen desaturation in patients with chronic obstructive pulmonary disease. Lung. 2013 Apr;191(2):177-82. doi: 10.1007/s00408-012-9443-3. Epub 2013 Jan 3.
57. Baldwin DR, Bates AJ, Evans AH, Bradbury SP, Pantin CF. Nocturnal oxygen desaturation and exercise-induced desaturation in subjects with chronic obstructive pulmonary disease. Respir Med. 1995 Oct;89(9):599-601. doi: 10.1016/0954-6111(95)90226-0.
58. Huang HY, Lo CY, Yang LY, Chung FT, Sheng TF, Lin HC, Lin CW, Huang YC, Chang CJ, Chung KF, Wang CH. Maintenance Negative Pressure Ventilation Improves Survival in COPD Patients with Exercise Desaturation. J Clin Med. 2019 Apr 25;8(4):562. doi: 10.3390/jcm8040562.
59. Martí S, Pajares V, Morante F, Ramón MA, Lara J, Ferrer J, Güell MR. Are oxygen-conserving devices effective for correcting exercise hypoxemia? Respir Care. 2013 Oct;58(10):1606-13. doi: 10.4187/respcare.02260. Epub 2013 Mar 19.
60. Delample D, Sabate M, Préfaut C, Durand F. Does prior training affect acute O₂ supply responses during exercise in Desaturator COPD patients? Open Respir Med J. 2008;2:29-34. doi: 10.2174/1874306400802010029. Epub 2008 Mar 13.
61. Watson K, Winship P, Cavalheri V, Vicary C, Stray S, Bear N, Hill K. In adults with advanced lung disease, the 1-minute sit-to-stand test underestimates exertional desaturation compared with the 6-minute walk test: an observational study. J Physiother. 2023 Apr;69(2):108-113. doi: 10.1016/j.jphys.2023.02.001. Epub 2023 Mar 11.
62. Poulain M, Durand F, Palomba B, Ceugniet F, Desplan J, Varray A, Préfaut C. 6-minute walk testing is more sensitive than maximal incremental cycle testing for detecting oxygen desaturation in patients with COPD. Chest. 2003 May;123(5):1401-7. doi: 10.1378/chest.123.5.1401.
